# Microbiome-driven identification of microbial indicators for postharvest diseases of sugar beets

**DOI:** 10.1186/s40168-019-0728-0

**Published:** 2019-08-07

**Authors:** Peter Kusstatscher, Christin Zachow, Karsten Harms, Johann Maier, Herbert Eigner, Gabriele Berg, Tomislav Cernava

**Affiliations:** 1 Austrian Centre of Industrial Biotechnology, Petersgasse 14, 8010 Graz, Austria; 2 Institute of Environmental Biotechnology, Graz University of Technology, Petersgasse 12, 8010 Graz, Austria; 3 Südzucker AG, Maximilianstraße 10, 68165 Mannheim, Germany; 4 Agrana Research & Innovation Center, Josef-Reither-Straße 21 – 23, 3430 Tulln, Austria; 5 Roombiotic GmbH, c/o: SciencePark, Stremayrgasse 16/IV, 8010 Graz, Austria

**Keywords:** *Beta vulgaris*, Storage rot, Indicator species, Phytopathogens, Bacterial microbiome, Fungal microbiome

## Abstract

**Background:**

Sugar loss due to storage rot has a substantial economic impact on the sugar industry. The gradual spread of saprophytic fungi such as *Fusarium* and *Penicillium* spp. during storage in beet clamps is an ongoing challenge for postharvest processing. Early detection of shifts in microbial communities in beet clamps is a promising approach for the initiation of targeted countermeasures during developing storage rot. In a combined approach, high-throughput sequencing of bacterial and fungal genetic markers was complemented with cultivation-dependent methods and provided detailed insights into microbial communities colonizing stored roots. These data were used to develop a multi-target qPCR technique for early detection of postharvest diseases.

**Results:**

The comparison of beet microbiomes from six clamps in Austria and Germany highlighted regional differences; nevertheless, universal indicators of the health status were identified. Apart from a significant decrease in microbial diversity in decaying sugar beets (*p* ≤ 0.01), a distinctive shift in the taxonomic composition of the overall microbiome was found. Fungal taxa such as *Candida* and *Penicillium* together with the gram-positive *Lactobacillus* were the main disease indicators in the microbiome of decaying sugar beets. In contrast, the genera *Plectosphaerella* and *Vishniacozyma* as well as a higher microbial diversity in general were found to reflect the microbiome of healthy beets. Based on these findings, a qPCR-based early detection technique was developed and confirmed a twofold decrease of health indicators and an up to 10,000-fold increase of disease indicators in beet clamps. This was further verified with analyses of the sugar content in storage samples.

**Conclusion:**

By conducting a detailed assessment of temporal microbiome changes during the storage of sugar beets, distinct indicator species were identified that reflect progressing rot and losses in sugar content. The insights generated in this study provide a novel basis to improve current or develop next-generation postharvest management techniques by tracking disease indicators during storage.

**Electronic supplementary material:**

The online version of this article (10.1186/s40168-019-0728-0) contains supplementary material, which is available to authorized users.

## Background

Plant-colonizing microorganisms live in close relationship with their host and are a crucial factor for plant growth and health [1–3]. For various crop plants, this was observed along the entire value-chain including the postharvest period [4]. The exploration of plant-microbe interactions, plant-beneficial bacteria and fungi including yeasts, their functions, and modes of action is a key for advanced developments related to biotechnological applications in agriculture [2, 5]. However, the development of postharvest applications based on biologicals is challenging due to the great diversity of postharvest pathogens as well as the often highly challenging postharvest treatments and storage conditions [6, 7]. The herbaceous dicotyledonous plant, *Beta vulgaris* L. (sugar beet) is the main crop for sugar production (sucrose content up to 18%) in temperate regions all over the world [8]. A number of plant pathogens such as *Pythium ultimum* Trow [9], *Rhizoctonia solani* Kühn [10], and *Cercospora beticola* Sacc. [11] cause severe harvest shortfalls due to seedling rot or late root rot [12]. After harvest, starting from late October, sugar beets are stored in Europe directly on the fields for a maximum of 60 days due to limited process capacities and increased economic viability of sugar refineries. High water (76%) and sugar content (18%) in the unprocessed beets [13] provide perfect conditions for microbial colonization, especially when cracks, root tip breakage, and fresh wounds on the surface provide easy entry points [14]. Microbial colonization, mainly by pathogenic or saprophytic fungi such as *Fusarium*, *Penicillium*, and *Botrytis* spp., leads to substantial sugar yield losses. A major observation is microbial inversion of sucrose into unwanted glucose and fructose molecules [15]. The combined occurrence of microbial degradation, respiration of the beet root, synthesis of raffinose, and other causes can yield sugar losses of up to 50–60% during storage [16, 17].

Natural antagonists that are part of the indigenous beet microbiome, previously studied by Zachow and colleagues (2008) [18], carry the potential for alternative plant protection applications during growth and postharvest [19, 20]. In our previous study, we found correlations between the disease incidence in sugar beet fields and the antagonistic potential of the prevalent microbiota [21]. These observations provide the basis for sustainable methods to prevent high sugar yield losses, caused by fungal infection with a targeted use of antagonistic microorganisms that could also provide postharvest protection [22]. However, in order to develop targeted and sustainable countermeasures, it is crucial to identify key players in the rot onset and to improve early detection strategies of rot-causing pathogens for beet clamps. Moreover, when biological control is employed, it is important to understand to which natural counterparts beneficial microorganisms will be exposed. Although rot-causing fungal pathogens were previously identified [14], the health-related dynamics of bacteria and fungi in stored sugar beets remained unexplored.

The aim of this study was to analyze temporal community changes in the microbiome of stored roots, correlate them to sugar beet health, and finally integrate the generated knowledge into a novel disease detection technique. Therefore, we investigated the bacterial and fungal microbiome of stored sugar beets in different beet clamps located in important cultivation areas of Austria and Germany. By implementing a detailed assessment of the beet clamp microbiome, specific biological markers indicating disease development in stored beets were found. These observations were thereafter confirmed with sugar beets stored under controlled conditions to verify the applicability of the identified markers. The overall findings provide a basis for novel postharvest management techniques that implement microbial and molecular markers for targeted countermeasures.

## Results

### Identification of fungal taxa from decaying sugar beets

**Fig. 1:**
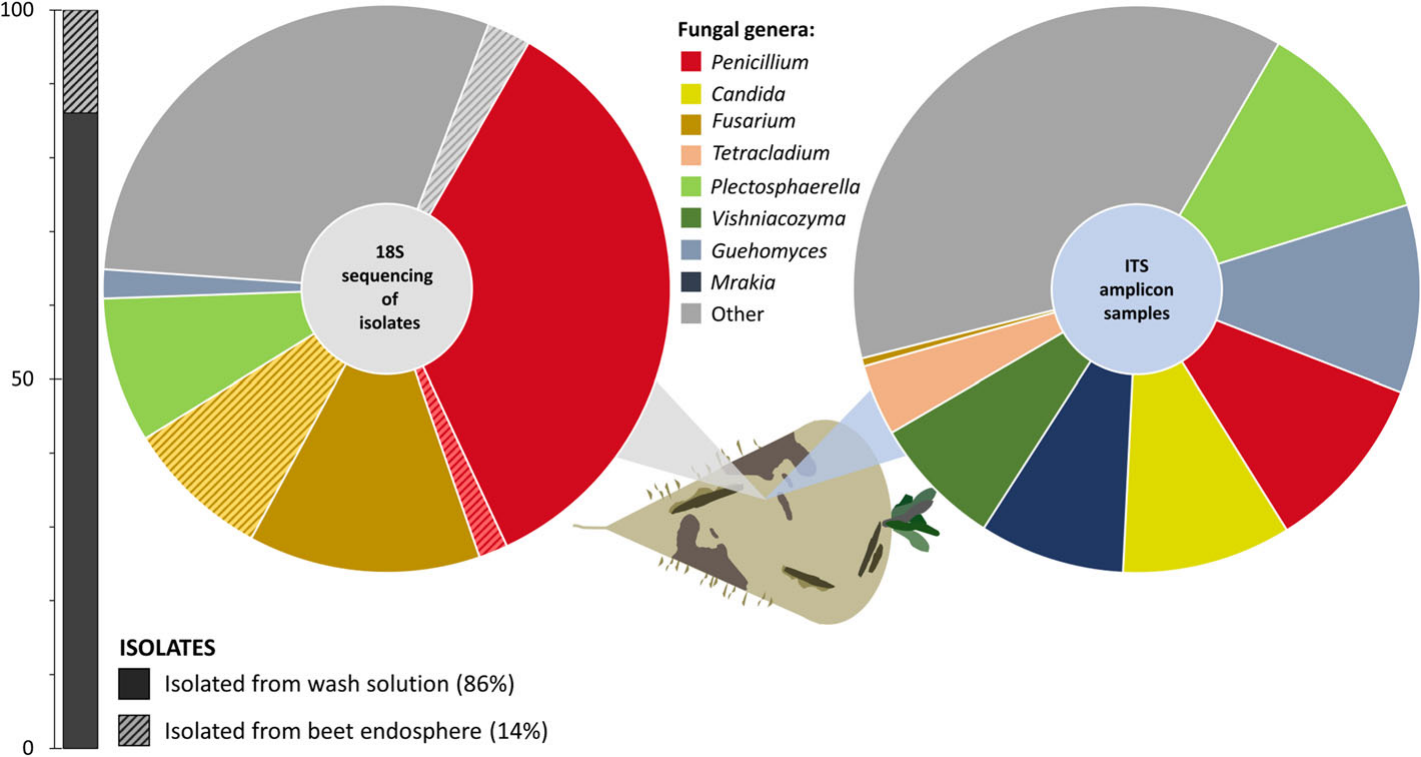
Comparison of 18S rRNA gene fragment sequencing results of fungal strains isolated from beet clamps in Austria and Germany and ITS sequencing of amplicon samples. The obtained sequences were assigned up to genus level. Color-coded segments indicate different genera in both datasets. Shaded areas represent the fraction of fungal isolates obtained from the beet endosphere

In order to identify fungal taxa in infected sugar beets from clamps in Austria and Germany, two complementary methods were applied. The community structure was reconstructed with Sanger sequencing of 18S rRNA gene fragments from fungal isolates and ITS Illumina amplicon sequencing of total community DNA (Fig. 1). The 18S rRNA gene sequencing-based community reconstruction with 120 fungal strains indicated a fungal community structure with 11 different genera, which was dominated by *Penicillium* (37%) and *Fusarium* (22%) species, while ITS amplicon sequencing indicated a more diverse composition. A total of 80 amplicon datasets revealed more than 50 different fungal genera. The most prominent genera were assigned to *Plectosphaerella* (11%), *Guehomyces* (10%), *Penicillium* (10%), *Candida* (10%), *Mrakia* (8%), *Vishniacozyma* (8%), and *Tetracladium* (4%). While *Penicillium* was abundant in both approaches, *Fusarium* was only predominant in the isolate-based community reconstruction. Moreover, the highest proportion of fungal strains (86%) was recovered from the beet surface; however, a substantial fraction of the identified *Fusarium* species (39%) originated from the sugar beet endosphere.

### Microbial diversity was significantly decreased in decaying sugar beets

**Fig. 2:**
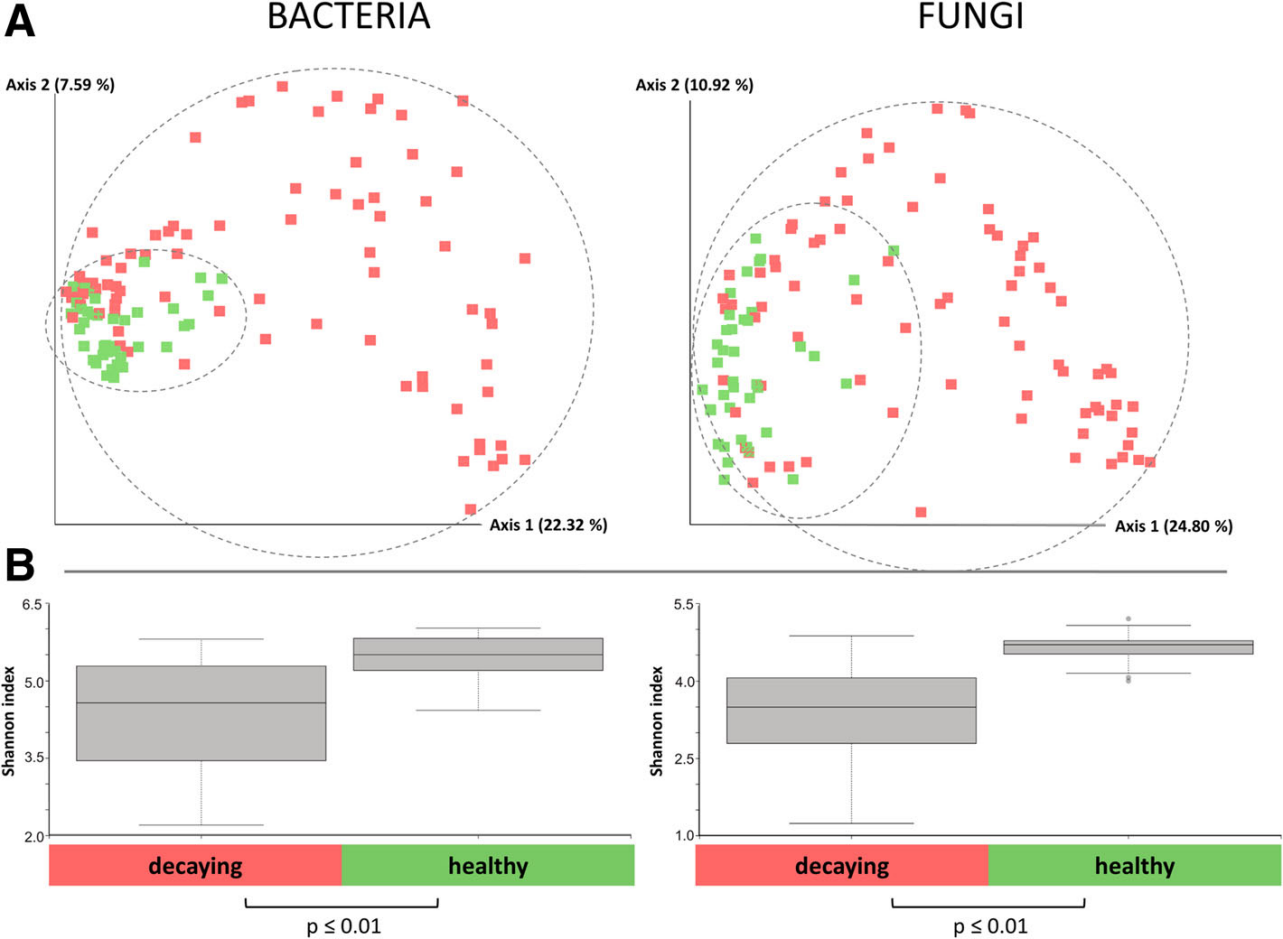
Alpha and beta diversity comparison of healthy and decaying sugar beet microbiome samples. The bacterial and fungal microbiome of each sample is indicated with one dot (**a**). Highly significant differences in the diversity were obtained from a total of 40 healthy and 80 decaying samples (**b**). Distances shown in the PCoA plot are based on the Bray Curtis diversity metrics

The comparison of amplicon data obtained from 120 samples of healthy and decaying sugar beets showed a significantly lower bacterial diversity in infected samples (Shannon index: 4.5 (16S) and 3.5 (ITS)) compared to the microbiome of healthy sugar beets (Shannon index 5.5 (16S) and 4.5 (ITS)) (Fig. 2b). The calculated Bray-Curtis distances showed significant differences in the composition of the microbiomes of the two groups. When a group-wise comparison was conducted, samples of decaying sugar beets (*n* = 80) clustered significantly (*p* value ≤ 0.01) different from samples of healthy sugar beets (*n* = 40). The variation within the infected group was found to be higher, compared to the healthy samples, which clustered more closely together (Fig. 2a).

### The core microbiome composition was altered in decaying sugar beets

**Fig. 3:**
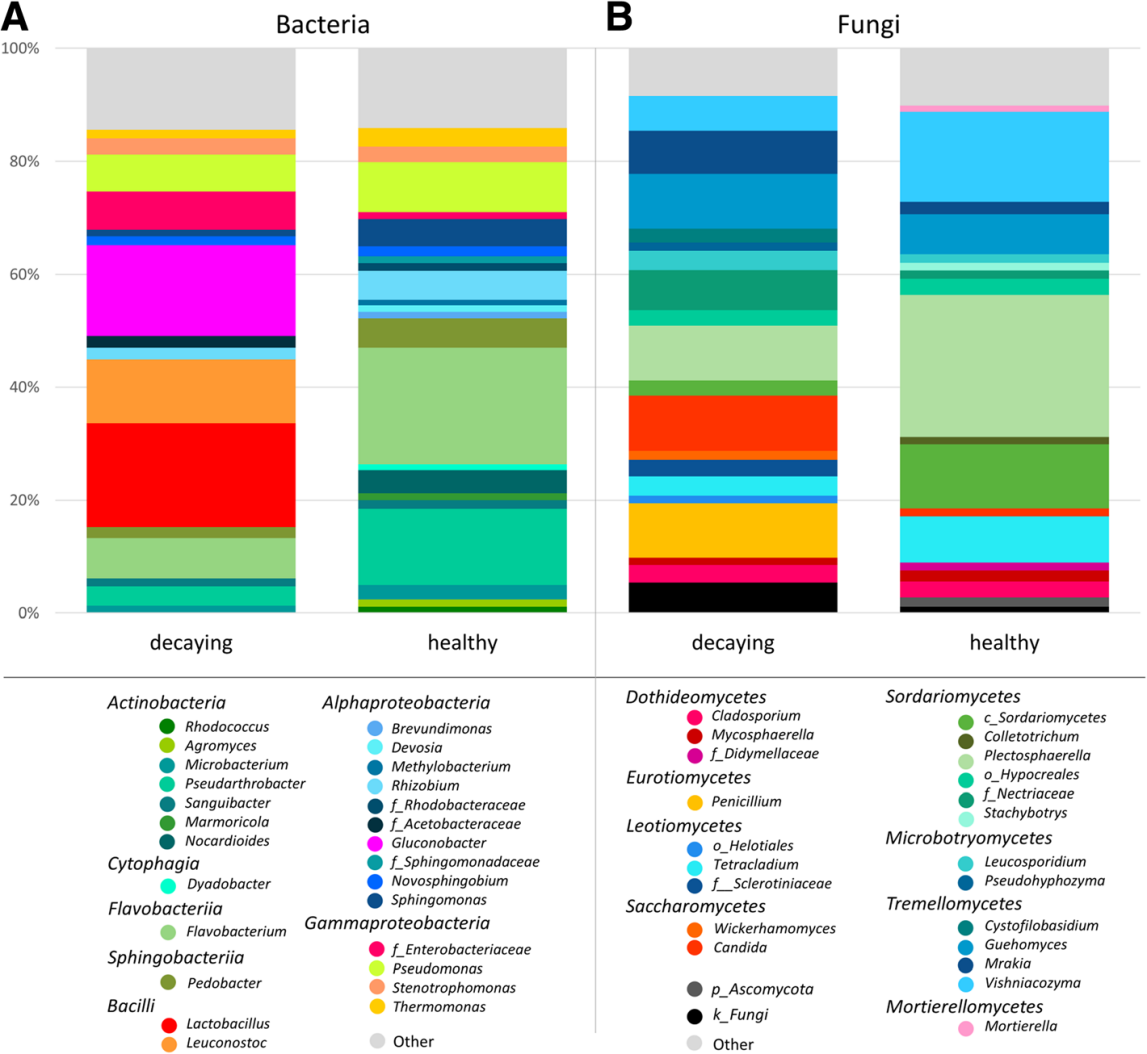
The core microbiome of healthy and decaying sugar beets from beet clamps in Austria and Germany. Relative abundances of prevalent bacterial (**a**) and fungal taxa (**b**) are shown. All taxa with an abundance ≥ 1% were identified on genus level if the resolution was sufficient. The grouping was conducted based on assignments at class level and taxa that were not assignable at genus level were additionally labeled: f_: family, o_: order, c_: class, p_: phylum, k_: kingdom

Taxonomic assignments of the identified features indicated a decay-specific microbiome of the analyzed sugar beets. The comparison of healthy and decaying samples showed a clearly distinguishable composition of taxa in both bacterial and fungal amplicon reads. *Proteobacteria* with an average relative abundance of 41% (healthy samples) and 51% (decaying samples) were the most abundant taxa on phylum level. *Bacteriodetes* (27% and 12.5%) and *Actinobacteria* (28% and 11%) were also highly abundant in both groups. The main difference between both groups was due to the phylum *Firmicutes* (0.4% in healthy and 25% in decaying samples). A major fraction of *Firmicutes* in the decaying samples belonged to the order of *Lactobacillales* (24%). The predominant *Proteobaceria* in healthy samples were mainly members of the orders *Pseudomonadales* (10%), *Sphingomonadales* (9%), *Rhizobiales* (8.5%), *Xanthomonadales* (6.5%), and *Enterobacteriales* (2.5%). In contrast, the 51% *Proteobacteria* found in decaying samples belonged to the orders *Rhodospirillales* (20%), *Enterobacteriales* (8%), *Pseudomonadales* (8%), *Xanthomonadales* (5%), *Sphingomonadales* (4%), and *Rhizobiales* (4%). At order level, the most abundant taxa of healthy sugar beets were *Flavobacteriales* (21%), *Micrococcales* (21%), and *Pseudomonadales* (10%), whereas the predominant taxa of decaying sugar beets were *Lactobacillales* (24%), *Rhodospirillales* (20%), and *Flavobacteriales* (9%). At genus level *Lactobacillus* (18.4%), *Gluconobacter* (16%), and *Leuconostoc* (11.3%) were the most abundant taxa in decaying samples, whereas *Flavobacterium* (20.6%), *Pseudarthrobacter* (13.5%), and *Pseudomonas* (9%) were the most abundant taxa in healthy samples. (Fig. 3a).

The ITS dataset showed diversified fungal microbiomes in both healthy and decaying sugar beets. When the structure of the whole dataset was assessed, a total of 60–62% *Ascomycota* and 33% *Basidiomycota* were observed within the fungal community. At class level, an increased fraction of *Saccharomycetes* (+ 10% points; 12% total) and *Eurotiomycetes* (+ 9% points; 10% total) as well as a decreased fraction of *Sordariomycetes* (− 16% points; 24% total) was found in the decaying samples. At order level, an increased abundance of *Cystofilobasidiales* (+ 11% points; 21% total), *Saccharomycetales* (+ 10% points; 12% total), and *Eurotiales* (+ 9.5% points; 10% total) was observed. At genus level, this resulted in an increased number of *Candida* (+ 7.5%; 9.5 total), *Penicillium* (+ 9.5%; 10% total), *Guehomyces* (+ 5%; 10% total), and *Mrakia* (+ 5%; 8% total). Healthy samples by contrast showed an increased amount of the genera *Plectosphaerella* (+ 10%; 21% total) as well as *Vishniacozyma* (+ 12%; 18% in total). This was already shown in an increased abundance of the classes *Sordariomycetes* (+ 16%; 40% in total) as well as *Tremellomycetes* (+ 2%; 30% in total). In comparison, at genus level, the most abundant genera in decaying samples were *Plectosphaerella*, *Guehomyces*, *Candida*, and *Penicillium* (all 10%), whereas in healthy samples the genera *Plectosphaerella* (21%) and *Vishniacozyma* (18%) dominated (Fig. 3b).

### Trophic specialization in the fungal microbiome

**Fig. 4:**
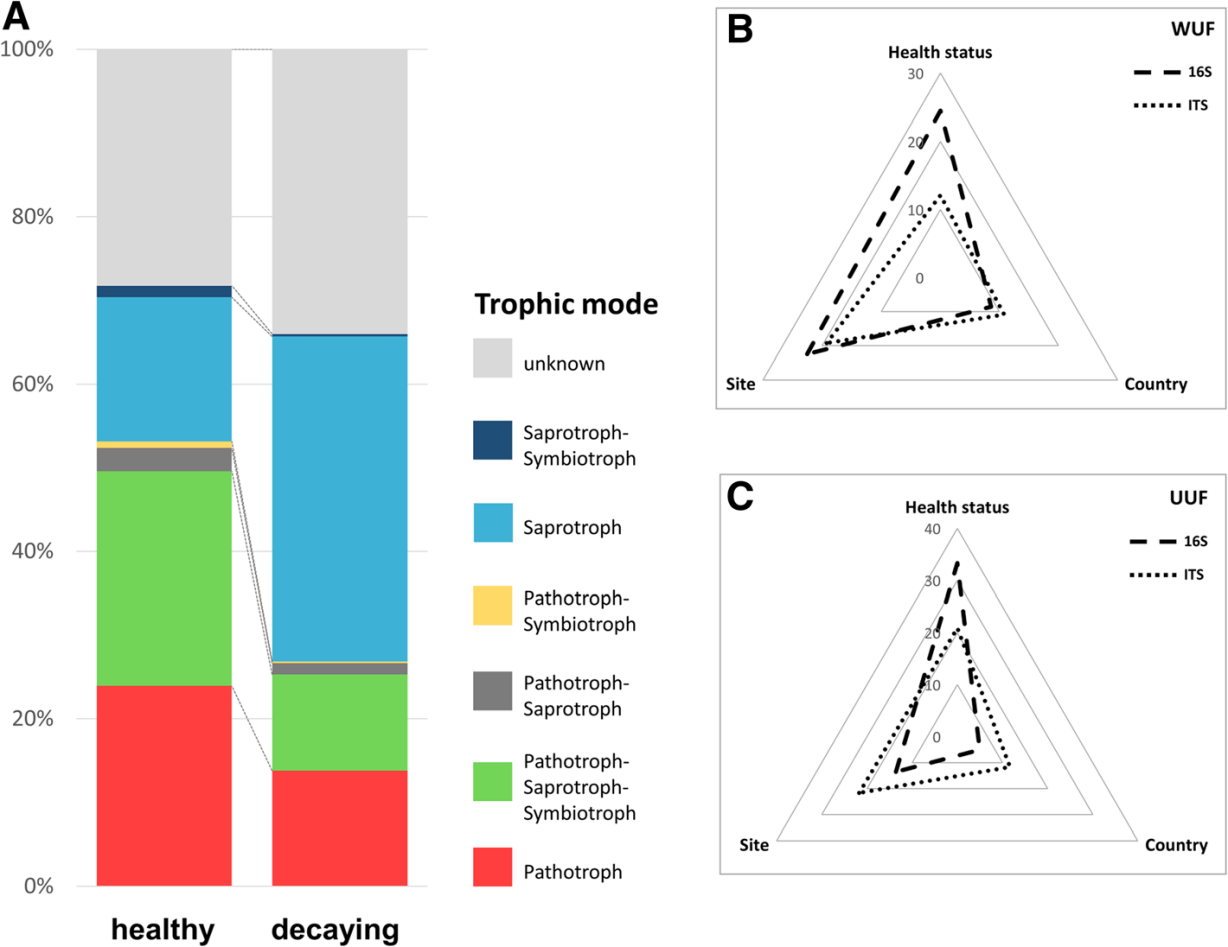
**a** Trophic modes in the fungal microbiome depending on sugar beet health status and **b**, **c** explained variance between samples by distinct parameters. The trophic modes were assigned according to identified core features of the samples and classifications stored in the FUNGuild database. A PERMANOVA analysis was performed by using weighted (WUF) as well as unweighted UniFrac (UUF) distance metrics

Taxonomic differences between healthy and decaying sugar beets were found to be accompanied by changes in the trophic modes of the identified core features. Healthy samples were mainly colonized by pathotrophic (24%) and pathotrophic-saprotrophic-symbiotrophic (26%) fungi. The trophic distribution in the decaying samples, however, was dominated by saprotrophic fungi (39%) with a decreased fraction of pathotrophic (14%) and pathotrophic-saprotrophic-symbiotrophic (12%) fungi. Overall, a decrease in pathotrophic and symbiotrophic functions and an increase in saprotrophic functions from the microbiome in healthy to the microbiome in decaying sugar beets was observed (Fig. 4a).

### The health status of beets was identified as the major driver for microbial community composition

The comparison of six different beet clamps in Austria and Germany showed significant differences in diversity as well as taxonomic composition. Health status explained the largest proportion of variance of the beets (33.3% variation in 16S dataset and 20.9% for ITS, *p* ≤ 0.001). Different beet clamp sampling sites also explained 13.6% variation in the 16S and 21.7% variation in the ITS dataset (*p* ≤ 0.001), however, variances within the groups were higher (*F* statistic = 3.43 (16S) and 6.25 (ITS) compared to 56.36 (16S) and 30.91 (ITS) between health statuses). The country that sugar beet samples originated from accounted for the least variance (5% in 16S data and 11.7% in ITS data, *p* ≤ 0.001) (Fig. 4b, c; Additional file 1: Table S1). These findings were also reflected in β-diversity PCoA plots, where sample were separated by health status (Additional file 1: Figure S1, S2).

**Fig. 5:**
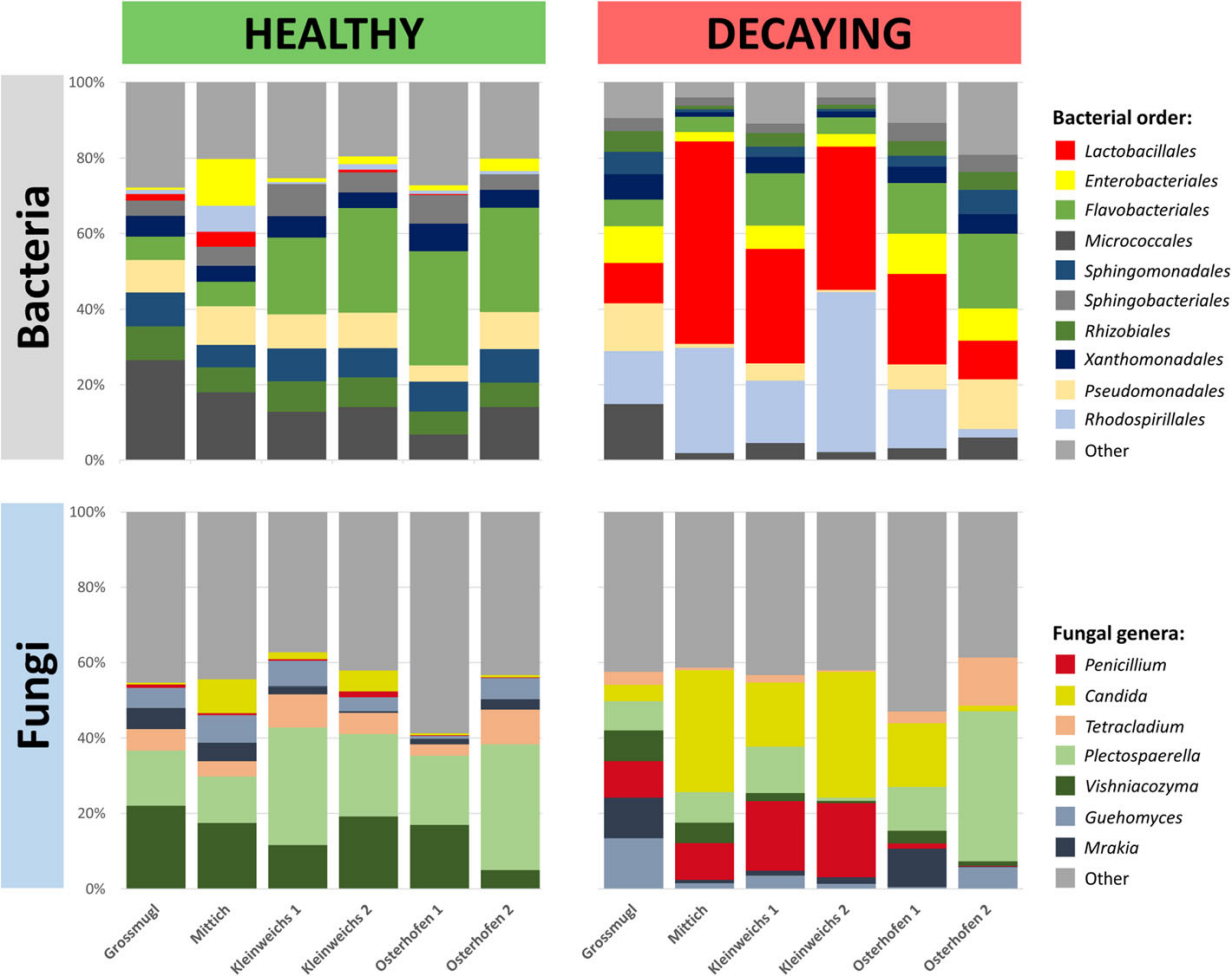
Relative abundance of the most abundant (≥ 5%) bacterial and fungal taxa in healthy and decaying sugar beet samples. Stored roots from six sugar beet clamps in Austria and Germany were analyzed by amplicon sequencing of the 16S rRNA gene fragment and the ITS region. The results were grouped according to the health status and the sampling site of the beets

Samples obtained from the storage in Grossmugl (Austria) showed clear differences in the microbial composition when compared to the sampling spots located in lower Germany (Mittich, Kleinweichs, and Osterhofen). Sampling locations that were geographically located closer to each other (Additional file 1: Figure S3C), however, showed less significant differences. Overall, a change from relatively balanced abundances of bacterial taxa (microbiome of healthy sugar beets) to a predominance of *Lactobacillales*, as well as *Rhodospirillales* (decaying sugar beets) was evident for every sampling spot. The fungal community changed from a microbiome dominated by *Vishniacozyma* and *Plectospaerella* to an increasing number of *Penicillium* and *Candida* species (Fig. 5).

### Identification of disease indicators and correlation to sugar content in stored sugar beets

**Fig. 6:**
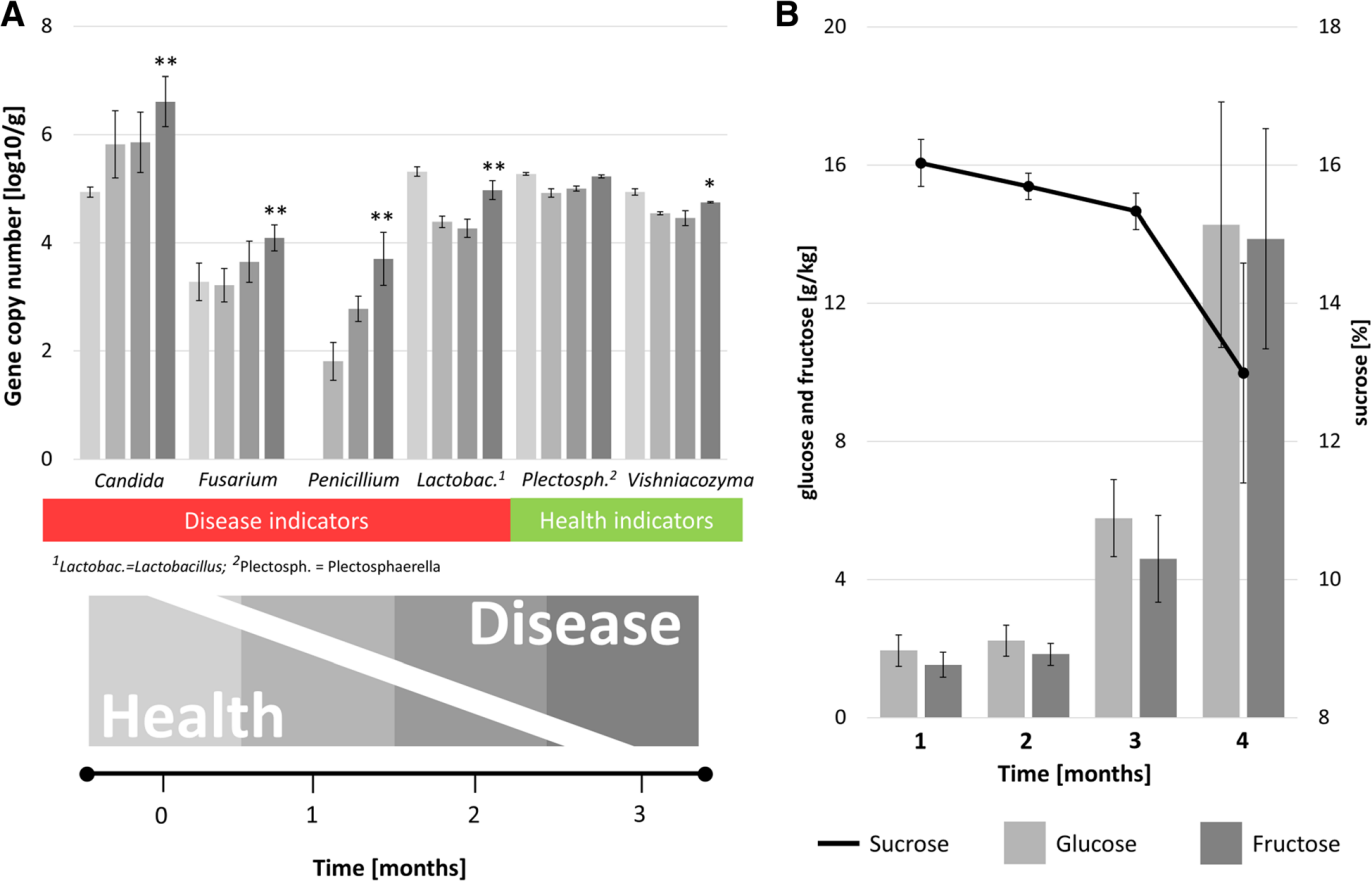
Real-time qPCR analysis of bacterial and fungal indicator taxa in stored sugar beets (**a**) and analyzed sucrose, glucose, and fructose contents in sugar beets (**b**). Gene copy numbers per gram sugar beet surface showed distinct tendencies related to accumulations of health and disease indicators during three months of controlled storage (color gradient). Statistical significance between the first and last measurement was tested using the Student’s *t* test: *p* value < 0.01 **; *p* < 0.05 *

Specific taxa, indicative either for the microbiome of healthy or decaying sugar beets, were selected based on the differences in their abundance in the representative samples (Figs. 3 and 5). *Flavobacterium* and *Pseudarthrobacter* within the bacterial community as well as *Plectospaerella* and *Vishniacozyma* within the fungal community were found to be dominant in healthy sugar beets. In contrast, *Lactobacillus* and *Gluconobacter* as well as *Candida* and *Penicillium* were prevalent in decaying sugar beets. By implementing a real-time qPCR analysis with specific primers targeting microbial indicators in stored sugar beets, the gradual increase of disease indicators and simultaneous loss of health indicators was shown. During a 3-month storage trial, an increase of *Candida* (10^5^ to 5 × 10^6^ copies/g), *Fusarium* (2 × 10^3^ to 10^4^ copies/g), and *Penicillium* (0 to 10^4^ copies/g) and simultaneous decrease of *Vishniacozyma* (10^5^ to 5 × 10^4^ copies/g) was observed (Fig. 6a). In case of *Plectosphaerella*, an initial decreases in abundance (2 × 10^5^ to 10^5^ copies/g), but overall constant abundances (10^5^ copies/g) throughout the storage period were found.

In order to verify the disease progress in the samples that were used for qPCR primer evaluations, complementary analytical analyses of beet carbohydrates were conducted with stored samples. The sugar content of sugar beets stored under controlled conditions showed a decreasing concentration of sucrose (− 3% points) during the storage period of 3 months. At the same time, an increase of inverted sugars (glucose (2 to 14 g/kg) and fructose (1.5 to 14 g/kg)) was observed (Fig. 6b).

## Discussion

### Fungal pathogens prevail among isolates from decaying sugar beets

The obtained results of the present study provide the first detailed microbiome characterization of conventionally stored sugar beets in an industrially scaled, uncontrolled environment. By combining different methods, a holistic assessment of the fungal microbiome in decay-affected sugar beets was constructed. 18S gene sequencing data of 120 fungal isolates was compared to ITS next-generation amplicon data. In comparison, 86% of isolated fungi identified on genus level were also found in the amplicon libraries. The cultivation-dependent identification of fungal isolates showed a prevalence of certain taxa such as *Penicillium* and *Fusarium*, when compared to the amplicon sequencing dataset. This likely resulted from the specific procedure during the isolation process that could have affected the frequency of isolated strains. While only homogenized peel was used for the total DNA extraction, also surface-sterilized fragments of infected sugar beets were placed on agar plates during isolation. This could have facilitated the isolation of *Fusarium* species, since this pathogen primarily colonizes the plant endosphere [23]. In the case of *Penicillium*, its high spore production allows it to overgrow slow-growing fungal taxa and likely lead to its isolation in higher proportions. The fungal ITS library obtained with high-throughput sequencing showed overall a higher diversity of different fungal taxa, which is partially due to cultivability limitations of certain taxa on standard isolation media [24, 25].

### Bacterial diversity decrease was accompanied by an emergence of several highly abundant lineages

Microbial diversity as well as distinct changes in the microbial community were previously shown to be linked to disease incidence [5, 26]. The data obtained in this study supports the hypothesis that lower diversity in the bacterial as well as fungal community is connected to a higher sensitivity to microbiome shifts that substantially alter the community structure. The lower diversity in decaying samples was reflected by a significant decrease in diversity indices in both the bacterial and the fungal dataset. In analogy to our findings, changed microbial diversity was found in stored onions when comparing healthy and diseased ones and fungal diversity was found to be higher in roots of healthy winter wheat plants [27, 28]. Moreover, a decrease in diversity was shown to facilitate invasion of pathogenic species into communities [29].

Even though amplicon-based sequencing can be affected by certain biases [30], the taxonomic composition of the bacterial as well as fungal beet microbiome, obtained with this dataset, was primarily linked to the health status of the sampled sugar beets. The geographic location of the beet clamps played a less significant role for the observed variability. Similarly, also Yurgel and colleagues (2018) observed taxonomic changes based on health status in stored onions [28]. Additionally, Liebe et al. (2016) already observed a similar effect in sugar beets when stored at different temperatures [14]. Depending on the storage conditions, the analyzed beets harbored specific fungal taxa, whereas the originating environment was less influential. In this study, sugar beets, stored under representative conditions without any protection from adverse environmental factors (moisture, temperature fluctuations, frost, etc.), showed a fungal community dominated by *Candida*, *Penicillium*, *Guehomyces*, and *Plectosphaerella* sp. in decaying sugar beets. The fungal microbiome of sampled healthy beet roots was, interestingly, comparable with the analyzed reference sugar beets in Liebe et al. (2016) harnessing mostly *Plectosphaerella* sp. [14]. The observed taxonomic changes were also reflected by trophic modes within the fungal community. Dominant pathotrophic and pathotrophic-saprotrophic-symbiotrophic functions in healthy samples were replaced by saprotrophic functions in decaying sugar beets. Similar findings were also made by Yu and colleagues (2012) linking the prevalence of saprotrophic fungi mostly to diseased pea plants, the abundance of pathogenic fungi, however, not to a specific health status [26].

### Identification of health indicators in the microbiome of sugar beets

Different potential biological markers were identified by contrasting healthy and diseased samples of stored sugar beets. Distinct taxa were shown to be highly abundant in samples representing each disease condition. The necrotrophic fungal lineage *Plectosphaerella*, found in healthy beets, was previously shown to be a growth-promoting microbe in sugar beets [31]. Moreover, it was reported as a potential biological control agent against potato cyst nematodes as well as a potential bioherbicide [32, 33]. Previous studies on sugar beet storage observed this taxon mostly in sugar beets before storage [14]. Other health-related taxa, such as *Flavobacterium* and *Pseudarthrobacter*, were often reported in the rhizosphere of different plants as well as their involvement in plant defense mechanisms or growth promotion [34–37]. Other taxa, associated with decaying sugar beets, such as *Penicillium*, are typical saprophytic fungi and postharvest pathogens and were observed previously in rotting sugar beet after harvest [14, 38, 39]. *Lactobacillus* as well as the fungal genus *Candida* were predominantly detected in decaying sugar beets and are associated with sugar fermentation to acid or alcohol compounds and are unwanted in stored sugar beets because of this activity [40, 41]. We hypothesize that such taxa occur on decaying sugar beets primarily due to increased free monosaccharides originating from the hydrolyzation processes of sucrose by fungal extracellular proteins.

Real-time qPCR analyses conducted on the basis of the identified health and disease indicators in stored sugar beets provided a first evidence for the applicability of such indicators for agricultural management strategies. The data was obtained within small-scale experiments and must be further expanded in upcoming approaches to confirm the reliability of the indicators for industry-scale applications. During the representative storage period of three months, health-related indicators were either decreasing or remained constant. In contrast, disease-related indicators increased substantially over the storage period. The quantitative analysis of these taxa indicated a gradual disease development that is linked to microbial sucrose concentration loss and simultaneously increase in inverted sugars during storage [42], which was confirmed by targeted analyses in the present study.

## Conclusion

Storage rot in stored sugar beets was shown to be accompanied by a change in microbial abundances. The present study highlighted substantial shifts within the bacterial as well as fungal community that correlated to decay incidence in stored roots. Changes in the prevalence of certain taxa can potentially indicate decay development at an early stage and facilitate an implementation of targeted countermeasures. Taxonomic changes were shown to be accompanied by trophic specialization in the fungal community. For upcoming postharvest applications, the novel insights provide a basis to design suitable biocontrol agents maintaining the balance of taxa associated with the microbiome of healthy sugar beets and preventing the establishment of degrading microorganisms. Furthermore, the identification of diseases indicators can be used as decision tool and supports the prioritization of processing of harvested beets during storage management. Additional studies are needed to confirm the implementability of the obtained results and to assign levels of quantitative measurements, which will allow to indicate the degree of disease.

## Methods

### Sampling of sugar beets and isolation of fungi

Healthy (*n* = 40) and decaying (*n* = 80) sugar beets were obtained from beet clamps in Austria (Upper Austria) and Germany (Bavaria). The detailed sampling locations are provided in Additional file 1: Table S2 and Figure S3C. Decaying sugar beets were obtained from nests of fungal mycelia in the beet clamps (Additional file 1: Figure S3A, B). Samples with severe and intermediate fungal infection were selected. Healthy sugar beets were collected from the non-infected, symptoms-free surrounding area of infected beet clamps. Following the sampling, 20 g of the sugar beet skin (surface of tap root and stem end) was peeled and washed with 50 mL of 0.85% sodium chloride solution in a stomacher (BagMixer; St. Nom, France) for 3 min. The obtained solution was prepared for total community DNA extraction as described later. A total of 100 μL of the solution obtained from decaying sugar beets was plated on SNA plates [43] containing penicillin G (100 μg/mL), dihydrostreptomycinsulfate (50 μg/mL), and chlortetracycline (10 μg/mL) in serial 1/10 dilutions until a final dilution of 10^−10^ was reached. In addition, surface sterilized (submerged in 4% sodium hypochlorite, 5 min) and washed (two times sterile distilled water) beet sections from diseased beets were placed on a SNA plate to obtain fungal isolates growing in the beet endosphere. A total of ten fungal strains per sugar beet were randomly picked based on morphology from the plates and further subcultured on PDA, SNA, and water agar plates (tap water + 18 g/L agar). The strains were further grouped using morphologic clustering after inspecting the single isolates on the different plates. Several strains of each morphologic cluster (120 strains in total) were subjected to 18S rRNA gene fragment Sanger sequencing (LGC Genomics, Berlin, Germany). Quality checked sequences were blasted against the NCBI database as well as the UNITE v7 database [44].

### Storage of sugar beets under controlled conditions

A total of 20 untreated and undamaged sugar beets harvested from a single field in Germany (Rhenish Hesse, Rhineland-Palatinate; 49° 35′ 54.388″ N, 8° 12′ 48.823″ E) were stored directly after harvest under controlled condition at 8 °C and 75% relative humidity for 3 months. Sampling of five sugar beets at the beginning (T0) and every 30 days (T1, T2, and T3) was performed as described above. A total of 20 g of sugar beet peel was washed in a stomacher with 50 mL of sodium chloride (0.85%). A total of 4 mL of the solution was centrifuged into a pellet and further used for community DNA extraction. Sugar content in the sugar beet flesh was measured using standardized ICUMSA (International Commission for Uniform Methods of Sugar Analysis) methods for the determination of glucose and fructose by enzymatic assays and the polarization of sugar (sucrose) by the cold aqueous digestion method [45, 46].

### Total community DNA extraction and construction of amplicon library

A total of 4 mL of the obtained washing solution from the sampling step was centrifuged (13,000×*g*, 20 min, 4 °C) and the pellet was stored at − 70 °C until further use. Using the FastDNA® Kit for Soil (MP Biomedicals/USA) genomic DNA was extracted from all samples. All steps were conducted as stated in the manufacturer’s protocol. Following DNA extraction, the 16S rRNA primers 514f and 926r (GTGYCAGCMGCCGCGGTAA; CCGYCAATTYMTTTRAGTTT) and the ITS primer pair ITS1f and ITS2r (CTTGGTCATTTAGAGGAAGTAA; GCTGCGTTCTTCATCGATGC) were used in PCR for amplicon library construction. As described in the protocols and standards section of the Earth microbiome project [47], both primer pairs were modified with specific primer pads (TATGGTAATT/AGTCAGCCAG) and linker (GT/GG) for the attachment of a Golay barcode sequences. Two consecutive PCR reactions were performed and all PCR reactions, conducted in triplicates were pooled after the second PCR. The first PCR (amplification of the V4 and V5 region or ITS1 region) was performed in a total volume of 10 μL (1 μL DNA, 2 μL Taq&Go, 0.1 μL of each Primer, 0.15 μL of mPNA and pPNA, and 6.5 μL of water). Added blocking primers mPNA and pPNA prevented the amplification of mitochondrial and chloroplast DNA [48]. The reactions were performed on a Whatman Biometra® Tpersonal and Tgradient thermocycler (Biometra GmbH, Göttingen, Germany) with the following settings: 95 °C for 45 s, 78 °C 5 s, 55 °C 45 s, 72 °C 90 s (35×), including an initial denaturation of 5 min at 95 °C and a final extension of 5 min at 72 °C. A second PCR step (multiplexing with Golay barcodes) a total volume of 30 μL (2 μL of the first PCR (template), 6 μL Taq&Go, 1.2 μL of barcode-primers and 19.6 μL of water) run at the following settings: 95 °C for 30 s, 53 °C 30 s, 72 °C 30 s (15×), including an initial denaturation of 5 min at 95 °C and a final extension of 5 min at 72 °C. After each PCR amplification step, the quality was checked by gel electrophoresis. All tree replicates of quality checked PCRs from each sample were pooled and purified using the Wizard SV Gel and PCR Clean-Up System (Promega, Madison, USA) according to the protocol. Equimolar DNA concentrations of each barcoded amplicon sample were sent to GATC Biotech AG, Konstanz, Germany. After entry quality control and adapter ligation, 16S rRNA and ITS gene amplicons were sequenced on an Illumina HiSeq instrument.

### Data evaluation using bioinformatics tools

Data obtained with Illumina HiSeq amplicon sequencing was analyzed with QIIME 2 (2018.6 release) and QIIME 1.9.1 [49] according to tutorials provided by the QIIME developers. After joining forward and reversed reads and barcode extraction in QIIME 1.9.1, the data was imported into QIIME 2 for further analysis. After demultiplexing, the DADA2 algorithm [50] was applied to denoise and truncate the reads and summarize sequence variants (SVs) in a feature table. To increase the quality, chimeric data was filtered as well as mitochondria and chloroplast reads (for 16S data) or bacteria and archaea reads (for ITS data) were discarded. A total of 3489 ITS and 8935 16S SVs were assigned for a total of 16,155,698 ITS and 4,036,955 16S reads (Additional file 1: Table S3). Alpha diversity, beta diversity, as well as statistical analysis was performed using the QIIME2 core diversity metrics. Naïve-Bayes classifier were trained on the SILVA v128 [51] at 99% similarity as well as the UNITE v7.2 [44] database for taxonomic assignment. Subsequently, core microbiomes (features present in at least 50% of the samples) were calculated for each group (healthy and decaying) and exported for display in bar charts. Functional analysis of fungal feature tables was performed using the FUNGuild online tool [52].

### Statistical analysis of bioinformatics data

Alpha and beta diversity was tested in QIIME 2. Therefore, the Kruskal-Wallis (alpha) and the anosim test (beta) were used. Variance explained by parameters was analyzed with a PERMANOVA test in QIIME. Significant taxonomic differences between the groups were observed with the ANCOM test in QIIME 2.

### Real-time qPCR measurement targeting microbial indicators

Following the community DNA extraction from stored sugar beet samples obtained under controlled conditions, qPCR amplifications using specific primers were conducted in order to quantify distinct taxonomic groups that were selected as disease indicators. Specific primers targeting *Candida*, *Fusarium*, *Penicillium*, *Lactobacillus*, as found in previous literature were implemented. Primers for *Vishniacozyma* and *Plectosphaerella* were designed using the Primer-BLAST tool [53] and deposited sequences in the NCBI database (Table 1). The quantification was performed with a Corbett Research TM thermocycler (Rotor-Gene 6000, Corbett Research, UK) and SYBR Green PCR master mix TM (KAPA Biosystems, USA). The standard curves were obtained using a single isolate gene fragment with known copy numbers and further 1:10 dilutions. Three replicates of each standard dilution were prepared to calculate mean values. The standards were employed to determine the gene copy numbers in the analyzed samples. Negative controls (using pure dH_2_O) were implemented and further subtracted from the analyzed samples to reduce quantification inaccuracies.

**Table 1:** Sequences, annealing temperatures, fragment length, and sources of the implemented qPCR primers. The primers for *Vishniacozyma* and *Plectospaerella* were designed with deposited sequences (accession numbers provided) in the NCBI database and the Primer-BLAST tool [53]

Taxonomic group	Forward primer	Reverse primer	Length (bp)	Annealing temp (°C)	Reference/source
*Vishniacozyma*	CGCATCGATGAAGAACGCAG	AAAACCCAAGTGGGGTGAGG	151	64.6	NR_073260.1, this study
*Plectospaerella*	ATCTCTTGGCTCCAGCATCG	GATACTGGAAGGCGCCATGT	112	65	GU724980.1, this study
*Candida*	TCTAACGTCTATGCGAGTG	ATACCCAAATTCGACGATCG	244	59.4	[54]
*Fusarium*	CAACTCCCAAACCCCTGTGA	GCGACGATTACCAGTAACGA	398	58	[55]
*Lactobacillus*	GCAGCAGTAGGGAATCTTCCA	GCATTYCACCGCTACACATG	342	62.1	[56]
*Penicillium*	ATGAAATCCTCCCTGTGGGTTAGT	GAAGGATAATTTCCGGGGTAGTCATT	92	65	[57]

## Additional file

Additional file 1: **Table S1.** Summary of performed PERMANOVA test. Pairwise comparison of categories using the unweighted (UUF) and weighted (WUF) UniFrac distance metrics for both, the 16S and ITS, datasets. Table S2: Sampling locations and sample conditions of the implemented sugar beets. Healthy and decaying beets were sampled from beet clamps in Austria (AT) and Germany (DE). At the locations Kleinweichs and Osterhofen, two neighboring beet clamps were sampled (1 and 2). Table S3: Overview of sequencing data. Number of reads, assigned sequence variants (SVs) using the DADA2 algorithm and Shannon Index of each group is given. Figure S1: Principal component analysis of bacterial and fungal communities from different beet clamps. PCoA using the unweighted UniFrac (UUF) distance metric. Samples are color-coded based on their geographic origin or health status. Figure S2: Principal component analysis of bacterial and fungal communities from different beet clamps. PCoA using the weighted UniFrac (WUF) distance metric. Samples are color-coded based on their geographic origin or health status. Figure S3: Sample visualization, schematic representation of fungal growth in the beet clamps, and geographic locations of the sampling sites. Fungal nests start within the clamp and spread to the surrounding beets (A, B). Healthy, uninfected beets, as well as decaying sugar beets within the same beet clamp were sampled from six different beet clamps in Austria and Germany (C). (DOCX 1605 kb)

## Abbreviations

DNA: Deoxyribonucleic acid
ITS: Internal transcribed spacer
PCR: Polymerase chain reaction
qPCR: Real-time (quantitative) polymerase chain reaction
