# Supplementary material for: Microbiome-driven identification of microbial indicators for postharvest diseases of sugar beets
Source: Microbiome. 2019 Aug 7;7:112. doi: 10.1186/s40168-019-0728-0 (PMC6686572; doi:10.1186/s40168-019-0728-0)
Supplement: Supplementary file 1 — Table S1. Summary of performed PERMANOVA test. Pairwise comparison of categories using the unweighted (UUF) and weighted (WUF) UniFrac distance metrics for both, the 16S and ITS, datasets. Table S2: Sampling locations and sample conditions of the implemented sugar beets. Healthy and decaying beets were sampled from beet clamps in Austria (AT) and Germany (DE). At the locations Kleinweichs and Osterhofen, two neighboring beet clamps were sampled (1 and 2). Table S3: Overview of sequencing data. Number of reads, assigned sequence variants (SVs) using the DADA2 algorithm and Shannon Index of each group is given. Figure S1: Principal component analysis of bacterial and fungal communities from different beet clamps. PCoA using the unweighted UniFrac (UUF) distance metric. Samples are color-coded based on their geographic origin or health status. Figure S2: Principal component analysis of bacterial and fungal communities from different beet clamps. PCoA using the weighted UniFrac (WUF) distance metric. Samples are color-coded based on their geographic origin or health status. Figure S3: Sample visualization, schematic representation of fungal growth in the beet clamps, and geographic locations of the sampling sites. Fungal nests start within the clamp and spread to the surrounding beets (A, B). Healthy, uninfected beets, as well as decaying sugar beets within the same beet clamp were sampled from six different beet clamps in Austria and Germany (C). (DOCX 1605 kb) [file 40168_2019_728_MOESM1_ESM.docx]

**Supplementary Material**

**Microbiome-driven identification of microbial indicators for postharvest diseases of sugar beets (*Beta vulgaris* L.)**

**Peter Kusstatscher^ab^, Christin Zachow^a^, Karsten Harms^c^, Johann Maier^c^,**

**Herbert Eigner^d^, Gabriele Berg^b^, and Tomislav Cernava^be^**

*^a^ Austrian Centre of Industrial Biotechnology, Petersgasse 14, A-8010 Graz, Austria*

*^b^ Institute of Environmental Biotechnology, Petersgasse 12, A-8010 Graz, Austria*

*^c^ Südzucker AG, Maximilianstraße 10, 68165 Mannheim, Germany*

*^d^ Agrana Research & Innovation Center, Josef-Reither-Straße 21 – 23, 3430 Tulln, Austria*

*^e^ Roombiotic GmbH, c/o: SciencePark, Stremayrgasse 16/IV, 8010 Graz, Austria*

**e-mail addresses:** peter.kusstatscher@acib.at; christin.zachow@acib.at; karsten.harms@suedzucker.de; johann.maier@suedzucker.de; herbert2.eigner@agrana.com; gabriele.berg@tugraz.at; tomislav.cernava@tugraz.at

**author of correspondence:**

Tomislav Cernava, Graz University of Technology, Institute of Environmental Biotechnology, Petersgasse 12, A-8010 Graz, email: [tomislav.cernava@tugraz.at](mailto:tomislav.cernava@tugraz.at), telephone: +43 316 873 8423

**Submitted to** **Microbiome**

# Running title: Postharvest sugar beet microbiome

Table S1: Summary of performed PERMANOVA test. Pairwise comparison of categories using the unweighted (UUF) and weighted (WUF) UniFrac distance metrics for both, the 16S and ITS, datasets.

| **UUF** |  |  |  |  |  |  |  |  |  |
| --- | --- | --- | --- | --- | --- | --- | --- | --- | --- |
| **16S** |  |  |  |  |  |  |  |  |  |
| **Group 1** | **Group 2** | **Df** | **Permutations** | **SumOfSqs** | **MeanSqs** | **p-value** | **F.Model** | **R2sqared** | **%Influence** |
| **healthy** | **diseased** | 1 | 999 | 2.846 | 2.846 | 0.001 | 56.358 | 0.333 | 33.3 |
| **Austria** | **Germany** | 1 | 999 | 0.425 | 0.425 | 0.001 | 5.911 | 0.050 | 5.0 |
| **6 beet clamp locations** | | 5 | 999 | 1.160 | 0.232 | 0.001 | 3.420 | 0.136 | 13.6 |
|  |  |  |  |  |  |  |  |  |  |
| **ITS** |  |  |  |  |  |  |  |  |  |
| **Group 1** | **Group 2** | **Df** | **Permutations** | **SumOfSqs** | **MeanSqs** | **p-value** | **F.Model** | **R2sqared** | **%Influence** |
| **healthy** | **diseased** | 1 | 999 | 1.805 | 1.805 | 0.001 | 30.912 | 0.209 | 20.9 |
| **Austria** | **Germany** | 1 | 999 | 1.009 | 1.009 | 0.001 | 15.477 | 0.117 | 11.7 |
| **6 beet clamp locations** | | 5 | 999 | 1.871 | 0.374 | 0.001 | 6.251 | 0.217 | 21.7 |
|  |  |  |  |  |  |  |  |  |  |
| **WUF** |  |  |  |  |  |  |  |  |  |
| **16S** |  |  |  |  |  |  |  |  |  |
| **Group 1** | **Group 2** | **Df** | **Permutations** | **SumOfSqs** | **MeanSqs** | **p-value** | **F.Model** | **R2sqared** | **%Influence** |
| **healthy** | **diseased** | 1 | 999 | 1.229 | 1.229 | 0.001 | 36.742 | 0.245 | 24.5 |
| **Austria** | **Germany** | 1 | 999 | 0.432 | 0.432 | 0.001 | 10.658 | 0.086 | 8.6 |
| **6 beet clamp locations** | | 5 | 999 | 1.125 | 0.225 | 0.001 | 6.314 | 0.225 | 22.5 |
|  |  |  |  |  |  |  |  |  |  |
| **ITS** |  |  |  |  |  |  |  |  |  |
| **Group 1** | **Group 2** | **Df** | **Permutations** | **SumOfSqs** | **MeanSqs** | **p-value** | **F.Model** | **R2sqared** | **%Influence** |
| **healthy** | **diseased** | 1 | 999 | 3.968 | 3.968 | 0.001 | 16.044 | 0.121 | 12.1 |
| **Austria** | **Germany** | 1 | 999 | 3.543 | 3.543 | 0.001 | 14.120 | 0.108 | 10.8 |
| **6 beet clamp locations** | | 5 | 999 | 6.322 | 1.264 | 0.001 | 5.375 | 0.192 | 19.2 |

Table S2: Sampling locations and sample conditions of the implemented sugar beets. Healthy and decaying beets were sampled from beet clamps in Austria (AT) and Germany (DE). At the locations Kleinweichs and Osterhofen, two neighboring beet clamps were sampled (1 and 2).

| **Beet clamp** | **Country** | **Location** | **healthy**  **samples** | **decaying**  **samples** |
| --- | --- | --- | --- | --- |
| Grossmugl | AT | 48° 29' 33.065''N,  16° 14' 11.77''E | 15 | 40 |
| Kleinweichs 1 | DE | 48° 45' 44.896''N,  12° 50' 30.328''E | 5 | 13 |
| Kleinweichs 2 | DE | 48° 45' 59.35''N,  12° 49' 50.905''E | 5 | 9 |
| Mittich | DE | 48° 26' 18.139''N,  13° 23' 1.755''E | 5 | 8 |
| Osterhofen 1 | DE | 48° 42' 58.522''N,  13° 1' 1.293''E | 5 | 6 |
| Osterhofen 2 | DE | 48° 42' 39.42''N,  13° 1' 21.604''E | 5 | 4 |

Table S3: Overview of sequencing data. Number of reads, assigned sequence variants (SVs) using the DADA2 algorithm and Shannon Index of each group is given.

| **Sample** | **No. samples** | **No. ITS reads** | **No. 16S reads** | **SVs**  **ITS** | **SVs 16S** | **Shannon Index ITS** | **Shannon Index 16S** |
| --- | --- | --- | --- | --- | --- | --- | --- |
| **Decaying** | 80 | 12,704,534 | 3,140,874 | 1,862 | 5,024 | 3.5 | 4.5 |
| **Healthy** | 40 | 3,451,164 | 886,081 | 1,627 | 3,911 | 4.5 | 5.5 |
| **Total** | 120 | 16,155,698 | 4,036,955 | 3,489 | 8,935 | 3.8 | 4.8 |


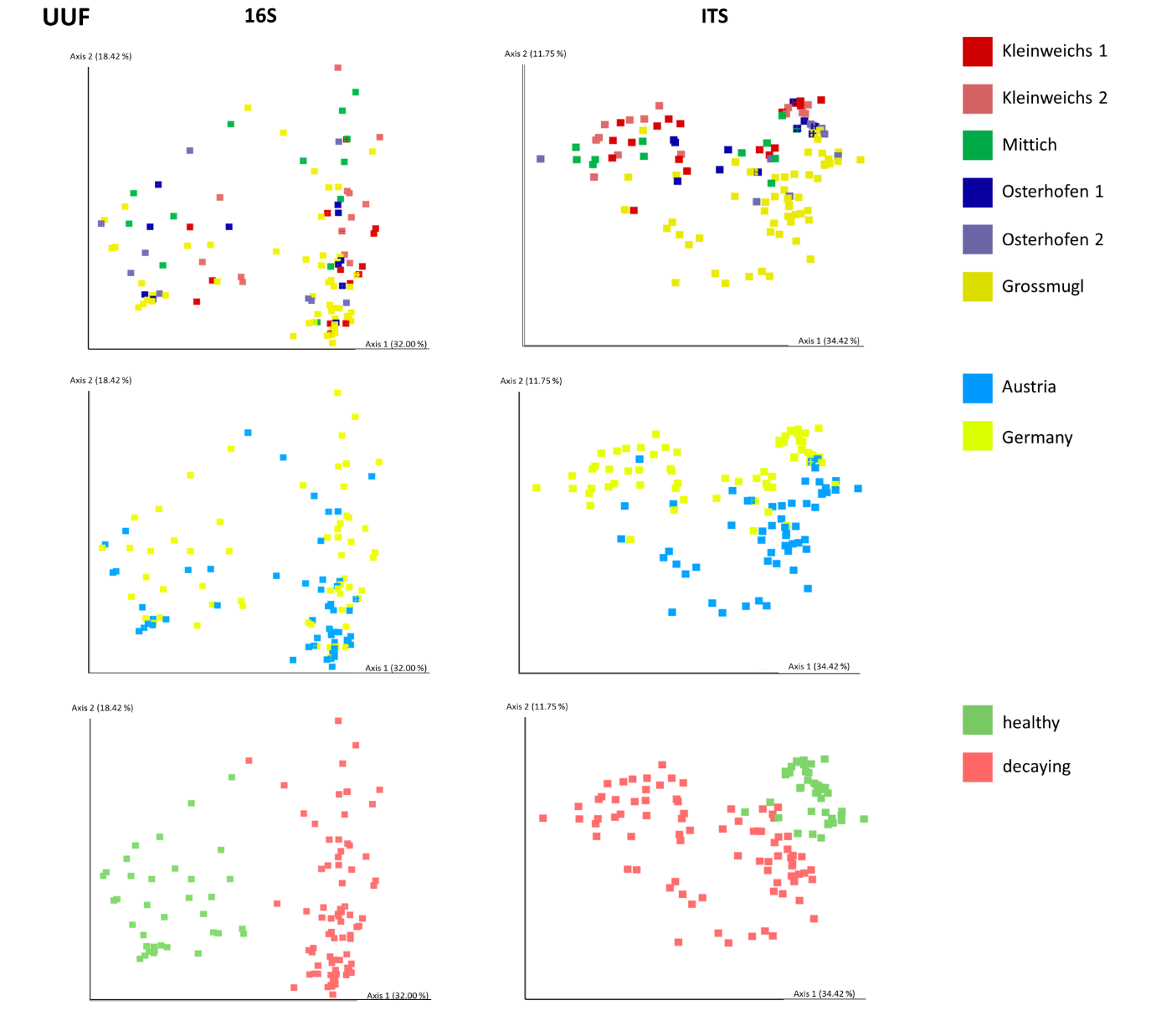


Figure S1: Principal component analysis of bacterial and fungal communities from different beet clamps. PCoA using the unweighted UniFrac (UUF) distance metric. Samples are color-coded based on their geographic origin or health status.


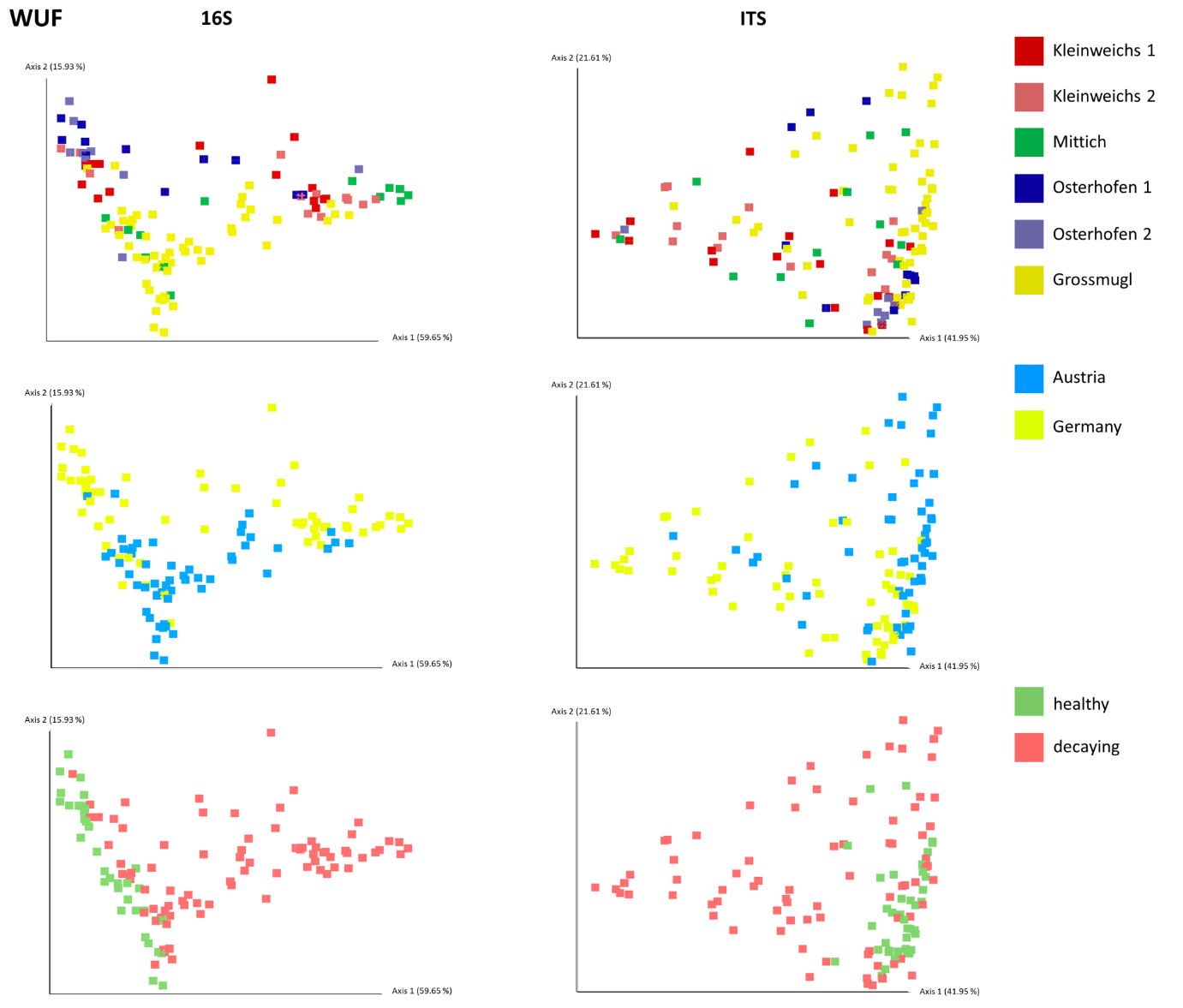


Figure S2: Principal component analysis of bacterial and fungal communities from different beet clamps. PCoA using the weighted UniFrac (WUF) distance metric. Samples are color-coded based on their geographic origin or health status.


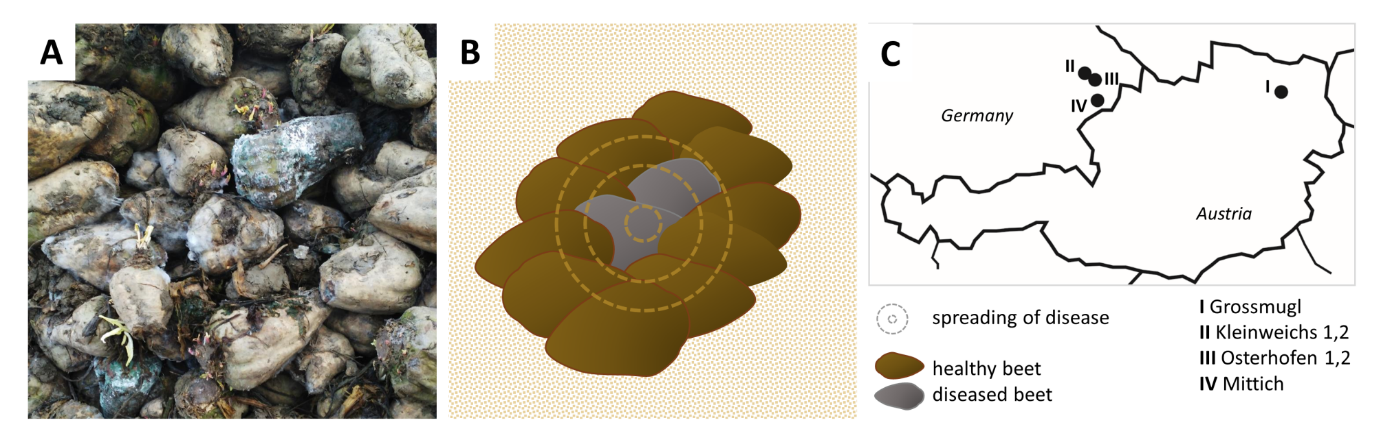


Figure S3: Sample visualization, schematic representation of fungal growth in the beet clamps, and geographic locations of the sampling sites. Fungal nests start within the clamp and spread to the surrounding beets (A, B). Healthy, uninfected beets, as well as decaying sugar beets within the same beet clamp were sampled from six different beet clamps in Austria and Germany (C).
